# 7p15 deletion as the cause of hand-foot-genital syndrome: a case report, literature review and proposal of a minimum region for this phenotype

**DOI:** 10.1186/s13039-017-0345-1

**Published:** 2017-11-15

**Authors:** Emiy Yokoyama, Dennise Lesley Smith-Pellegrin, Silvia Sánchez, Bertha Molina, Alfredo Rodríguez, Rocío Juárez, Esther Lieberman, Silvia Avila, José Luis Castrillo, Victoria del Castillo, Sara Frías

**Affiliations:** 1 Departamento de Genética Humana, Instituto Nacional de Pediatría, Insurgentes Sur 3700-C, Colonia Insurgentes Cuicuilco, Coyoacán, Ciudad de México, CDMX, Mexico; 2 Laboratorio de Citogenética, Departamento de Investigación en Genética Humana. Instituto Nacional de Pediatría, Avenida IMAN no. 1, Torre de Investigación, Colonia Insurgentes Cuicuilco, Coyoacán, Ciudad de México, CDMX, Mexico; 3 Laboratorio de Genética y Cáncer. Departamento de Genética Humana, Instituto Nacional de Pediatría, Avenida IMAN no. 1, Torre de Investigación, Colonia Insurgentes Cuicuilco, Coyoacán, México D.F, Mexico; 4 Laboratorio GENETADI, Bilbao, Spain; 5 Departamento de Medicina Genómica y Toxicología Ambiental. Instituto de Investigaciones Biomédicas, UNAM, Avenida IMAN no. 1, Torre de Investigación, Colonia Insurgentes Cuicuilco, Coyoacán. Ciudad de México, CDMX., México D.F, Mexico

**Keywords:** Hand-foot-genital syndrome, *HOXA13*, 7p15 deletion

## Abstract

**Background:**

Hand-foot-genital syndrome (HFGS) is a rare condition characterized by congenital malformations in the limbs and genitourinary tract. Generally, this syndrome occurs due to point mutations that cause loss of function of the *HOXA13* gene, which is located on 7p15; however, there are some patients with HFGS caused by interstitial deletions in this region.

**Case presentation:**

We describe a pediatric Mexican patient who came to the Medical Genetics Department at the National Institute of Pediatrics because he presented with genital, hand and feet anomalies, facial dysmorphisms, and learning difficulties. Array CGH reported a 12.7 Mb deletion that includes *HOXA13*.

**Conclusions:**

We compared our patient with cases of HFGS reported in the literature caused by a microdeletion; we found a minimum shared region in 7p15.2. By analyzing the phenotype in these patients, we suggest that microdeletions in this region should be investigated in all patients with clinical characteristics of HFGS who also present with dysplastic ears, mainly low-set implantation with a prominent antihelix, as well as a low nasal bridge and long philtrum.

## Background

Hand-foot-genital syndrome (HFGS; OMIM #140000) is an autosomal dominant condition that has been reported in 23 families [1–8] and 5 sporadic cases [9–12]. It is characterized by congenital limb malformations, mainly affecting the hands, such as short thumbs, and feet, as well as the genitourinary tract, such as hypospadias and cryptorchidism in males or urogenital septum and bicorne uterus in females [13]. HFGS is produced by mutations that cause loss of function of the *HOXA13* gene, which is located on 7p15 and is important for the development of fetal limbs and the urinary tract. The mutations responsible for HFGS include missense mutations, nonsense mutations, polyalanine expansions, and in very few cases, interstitial deletions at 7p15. We describe a Mexican patient with HFGS caused by a 12.7 Mb deletion on chromosome 7, ranging from p15.3 to p14.3, including the *HOXA13* gene. We compared previously reported patients with the purpose of determining a minimum shared region, as well as clinical characteristics that could guide the diagnosis of HFGS caused by a microdeletion.

## Case presentation

The patient is a 14-year-old male, the third child of a 21-year-old mother and a 30-year-old father, both of whom are healthy and non-consanguineous. He has two healthy brothers aged 20 and 18 years old. He was born after an uneventful pregnancy; his birth weight was 2300 g (<3th percentile), and his height was 49 cm (>15th percentile).

**Fig. 1:**
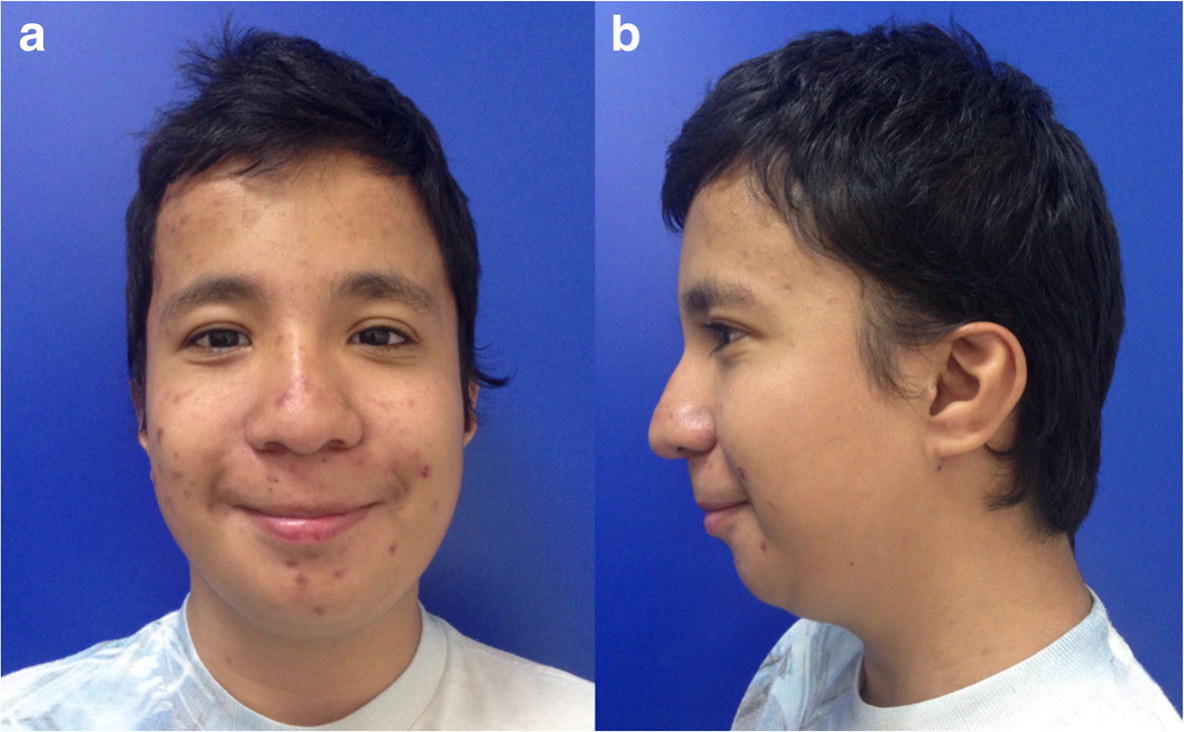
**a**. Facial phenotype of patient with midface hypoplasia, broad and prominent front, mild hypertelorism, strabism, broad nasal bridge, long and smooth philtrum, thin upper lip. **b**. Posteriorly rotated ears and prominent antihelix

**Fig. 2:**
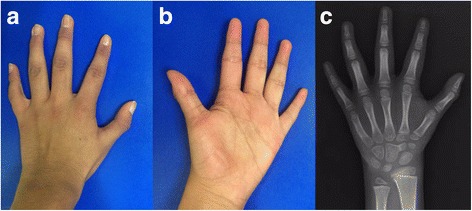
**a**. Small hands with pointed fingers, brachydactyly, bilateral fifth finger clinodactyly. **b**. Abnormal creases are observed. **c**. Small distal phalanges, metacarpal shortening

**Fig. 3:**
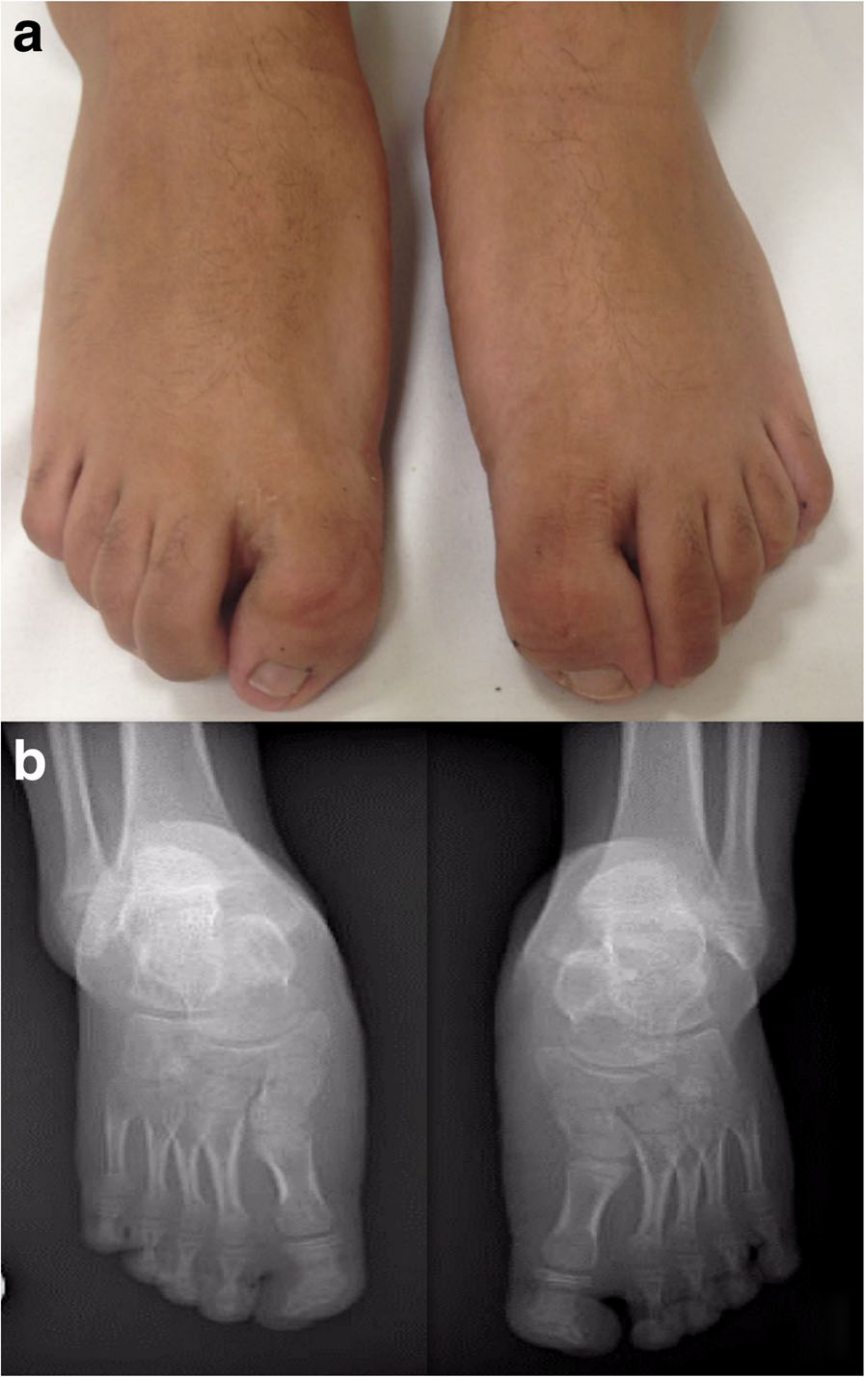
**a**. Feet with brachydactyly and severe bilateral hallux valgus. **b**. Metatarsal shortening and hypoplastic phalanges of all toes. Most notable is the thumb, which presents with valgus deviation of the last phalange

At 7 years old, the patient was referred to our hospital due to genital anomalies. Upon admission, a global developmental delay was detected, which was most profound in the area of language. He also presented with short stature, microcephaly, facial dysmorphisms, such as facial asymmetry, midface hypoplasia, a broad and prominent forehead, mild hypertelorism, strabismus, a broad nasal bridge, a long and smooth philtrum, a thin upper lip, a high palate (Fig. 1a), and low-set ears with a prominent antihelix (Fig. 1b). Additionally, we observed subglandular hypospadias and cryptorchidism, hands with fifth finger clinodactyly, abnormal palmar creases, and severe bilateral hallux valgus (Fig. 2a, b and 3a).

**Fig. 4:**
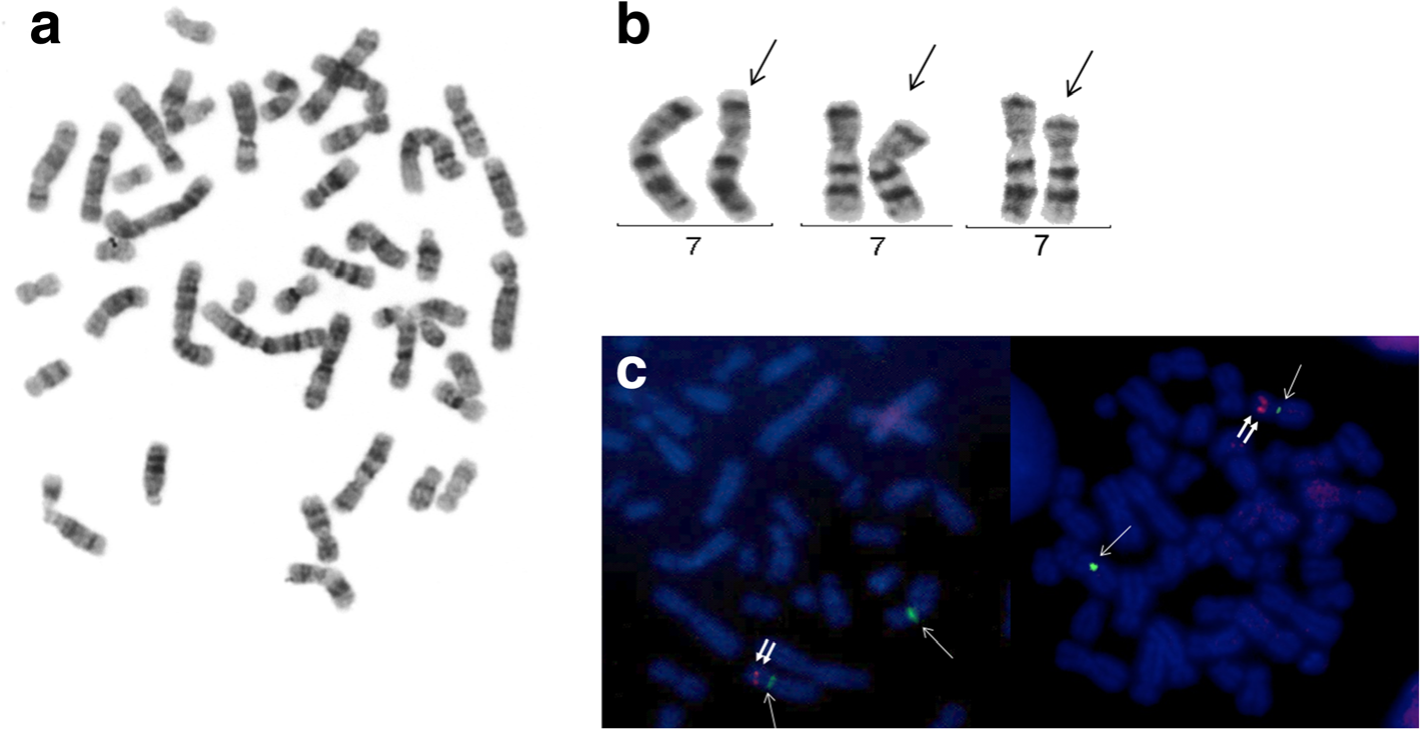
**a**. Karyotype with G-banding. **b**. Normal and deleted chromosome 7 (arrows) from different metaphases; **c**. FISH for the 7p15 region on metaphase chromosomes; alpha satellite probe for chromosome 7 centromere (D7Z1) marked with green fluorochrome Q-BIOgene (one thin arrow), and locus specific probe for 7p15.2 (where the *HOXA6-HOXA13* genes are normally located) marked with red fluorochrome-SureFiSH, Agilent (two thick arrows). Note that deleted chromosomes only have the green fluorescent probe and are missing the red fluorescent probe

**Fig. 5:**
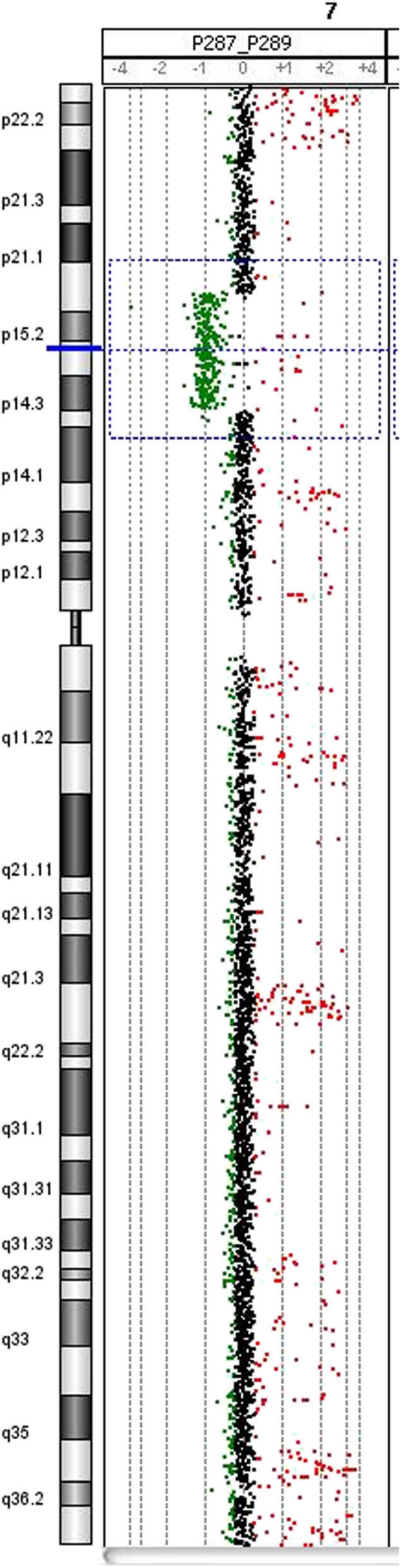
Array CGH of the patient. Array Agilent SurePrint G3 Human CGH microarray kit, 8x60K 46,XY.array [GRCh37/hg19] 7p15.3p14.3(22574164_35288260)x1dn (12,714,097 bp)

The patient’s brain MRI was normal, while auditory evoked potentials revealed normal left hearing and right conductive superficial hearing loss (secondary to serous otitis media). An X-ray of the hands showed a three-year delayed bone age, feet with an absence of mid-foot bone structures, such as cuneiform bones, only cuboid and navicular bones were observed, as well as bilateral hallux valgus, which was surgically repaired at 13 years old (Figs. 2c and 3b). Karyotype and Array CGH *Agilent 60 K* reported a 12.7 Mb deletion at 7p14.2p15.3 (Fig. 4a and b; ISCN 2016), which was confirmed by FISH with a specific 54.95Kb probe targeted to 7p15.2 inside the deleted region (Fig. 4c). The specific nomenclature is as follows: 46,XY.ish del(7)(p15.2)(D7Z1+,HOXA6−,HOXA13−).arr[GRCh37/hg19] 7p15.3p14.3(22574164_35288260)x1dn (Fig. 5; ISCN 2016). Both parents were normal. Analysis of the genomic alterations and the genotype-phenotype correlation enabled a diagnosis of hand-foot-genital syndrome.

## Discussion and conclusions

### Clinical aspects of hand-foot-genital syndrome

Some of the most frequent limb anomalies in HFGS are short thumbs, medial phalanges, small feet and clinodactyly of the fifth finger [13]. Hallux valgus is an important diagnostic sign, when present [11]. These anomalies have complete penetrance, but they can be so mild that imaging studies may be necessary to observe them. Short phalanges and pointed thumbs, hypoplasia of the first metacarpus and metatarsus, a small calcaneus, trapezium-scaphoid fusion, and cuneiform-navicular fusion can be observed radiologically [10]. Occasional findings include fusion of the medial and distal phalanges to the second to fifth toes [14], as well as delayed ossification [8].

It is estimated that half of the affected patients show urogenital malformations with variable expressivity. Male patients show hypospadias and cryptorchidism, while women present with a double uterus and partial or complete longitudinal urogenital septum. In the presence of any of these findings, HFGS must be investigated due to a 0.5–4% prevalence of the fusion of müllerian structures in the general population [8]. Fertility is conserved in HFGS, though there is an increased risk of spontaneous abortion, preterm birth, and stillbirth [2, 10, 11, 13].

In both genders, common findings include chronic urinary tract infections, ectopic ureteric orifices, vesicoureteral reflux, and pelviureteric junction. Additionally, the disease is associated with complications, such as chronic pyelonephritis and renal failure, which may require transplant. Psychomotor development and cognitive abilities are within normal range [11].

Differential diagnosis to be considered are Fanconi anemia syndrome, Rothmund Thomson syndrome, Holt-Oram syndrome, SALL4-related disorders, Nager syndrome, Townes-Brocks syndrome, and Lacrimo-auriculo-dento-digital (LADD) syndrome, due to the thumb hypoplasia. Incomplete Müllerian fusion and/or longitudinal vaginal septum should be cause for consideration of acro-renal-mandibular, Bardet-Biedl, Beckwith-Wiedemann, Fraser, Fryns, Halal, and Meckel syndromes. Heterozygous mutations in the *HOXA13* gene cause HFGS, however the specific c.1112A > T (p.Gln371Leu) mutation causes Guttmacher syndrome which shares some, but not all, features of HFGS and includes postaxial polydactyly of the hands, preaxial hand and foot deficiencies, short second toes, and glandular hypospadias [14]. Moreover, when patients present with short or absent nails and absent or hypoplastic distal phalanges without genital anomalies, brachydactyly type B1 and Cooks syndrome, must be considered [14].

### Genetics of HFGS

HOX genes are evolutionarily conserved; they participate in developmental processes and maintain their expression until adulthood [14]. They are organized into four clusters, *HOXA*, *HOXB*, *HOXC*, and *HOXD*, localized in 7p15.2, 17q21.32, 12q13.2, and 2q31.1, respectively. They contain between 9 and 11 genes each and codify transcription factors essential for morphogenesis [2]. In particular, *HOXA13* has 2 exons: exon 1 contains five polyalanine regions, and exon 2 has a homeobox domain that is involved in the development of distal limbs and the fetal urogenital tract [12]. *HOXA13* was proposed as candidate gene for HFGS and its involvement in limb development was corroborated in murine models [15].

The first identified pathogenic variant that causes HFGS in humans was the nonsense mutation c.1107G > A (p.Trp369Ter) [15]. Since then, there have been reports of pathogenic variants in both familial and de novo cases, including missense mutations (p.Arg326Gly, p.Asn372His, p.Gln371 Leu), nonsense mutations (p.Ser1360Ter, p.Gln1960Ter, p.Gln3650Ter and p.Trp3690Ter), and triplet expansions that code for a polyalanine region (6, 8–12, and 14 additional polyalanines) [7, 12]*.* Approximately 35% of these variants are localized within the coding region and, of these, 50 to 60% are found in the polyalanine regions [13, 15]. The missense pathogenic variants produce a gain of function mechanism associated with a more severe phenotype, while the nonsense mutations and polyalanine expansion variants are responsible for loss of protein function, presenting a milder phenotype [15].

### Microdeletions in HFGS

**Fig. 6:**
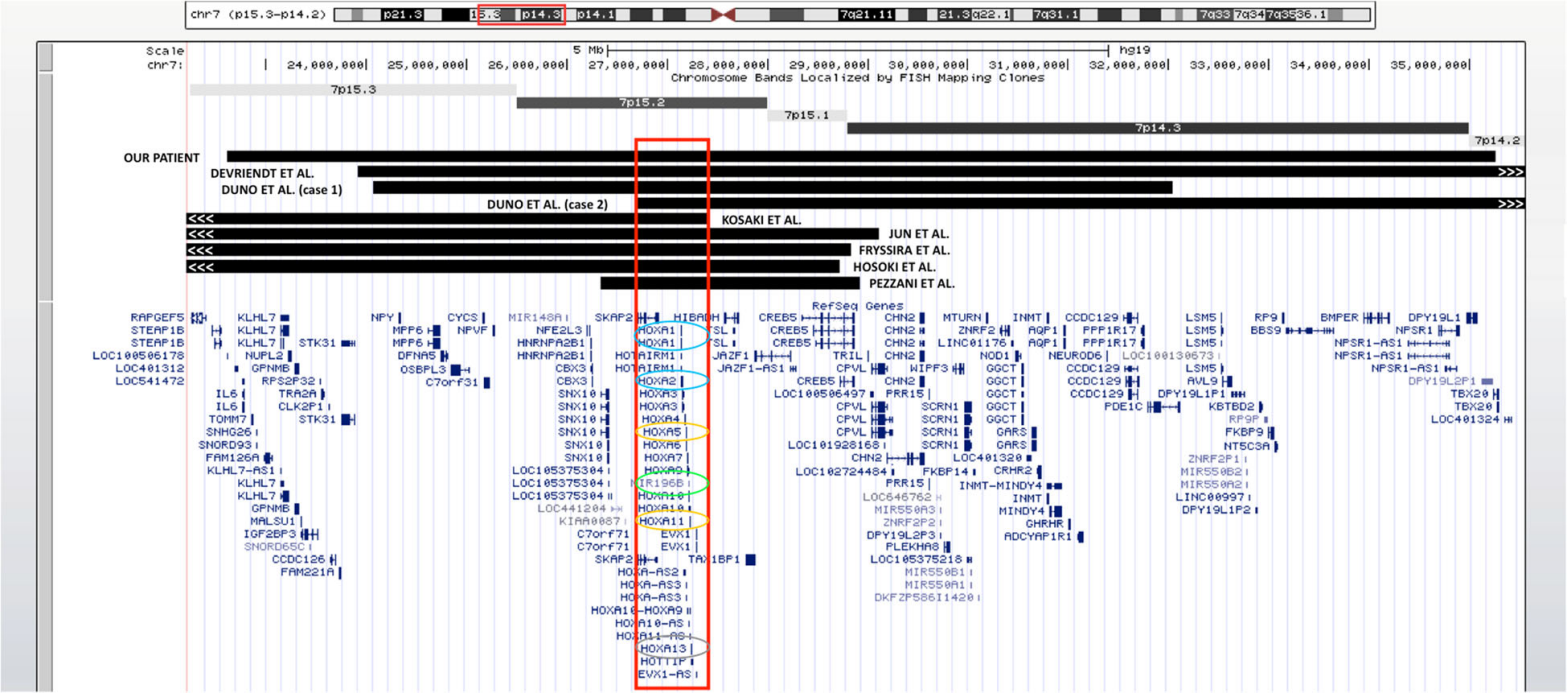
Genome Browser image comparing HFGS cases by microdeletion, included in Table 1. The red rectangle demarcates the minimum region (including the HOXA cluster) proposed as responsible for the phenotype of these patients. The most important genes that correlate with the proposed phenotype are: the *HOXA13* gene (gray circle), the *HOXA1* and *HOXA2* genes (blue circle), the *HOXA5* and *HOXA11* genes (orange circle), and miRNA196b (green circle)

Interstitial deletions including the 7p15 region are a rare cause of hand-foot-genital syndrome. To date, there are few reported patients with de novo deletions, which mostly occur between 7p15 and 7p21, with a variable size ranging from 2.5 to 12.7 Mb [9, 16–21]. We compared previously reported patients in the literature according to the genetic cause of the syndrome, polyalanine expansions, point mutations, and microdeletions; our patient was included in the latter group (Table 1). We found clinical features in patients with microdeletions that are not shared with patients carrying point mutations or those with polyalanine expansions (data not shown). Likewise, we found a minimum deleted region of 584,758 bp located at 7p15.2 that is shared by patients with HFGS. This region contains the *HOXA* cluster, the *SKAP2* and *EVX1* genes, and miRNA196b (Fig. 6).

**Table 1:** Clinical features of patients with deletion including 7p15 region and point mutations in *HOXA13*

	Patients with microdeletion in 7p15 region									Patients with point mutations in *HOXA13*			
	Patient 1 Present case	Patient 2 [9] (Devriendt et al., 1999)^c^	Patient 3 [18] (Dunø et al., 2004)^b^	Patient 4 [18] (Dunø et al., 2004)^b^	Patient 5 [19] (Kosaki et al., 2005) ^c^	Patient 6 [20] (Jun et al., 2011)	Patient 7 [21] (Fryssira et al., 2011)	Patient 8 [17] (Hosoki et al., 2012)	Patient 9 [16] (Pezzani et al., 2016)	Patient A [2] (Mortlock & Innis, 1997)	Patient B [3] (Goodman et al., 2000)	Patient C [10] (Parker et al., 2011)	Patient D [12] (Imagawa et al., 2014)
Chromosomal region (size) or mutation in *HOXA13* gene	7p14.3p15.3 (12.7 Mb)	7p14.2p15.3 (?)	7p14.3p15.3 (9.8 Mb) case 1	7p14.2p15.2 (9.0 Mb) case 2	7p15.2p21 (?)	7p15.1p15.3 (5.6 Mb)	7p14.3p21.1 (13 Mb)	7p15.1p15.3 (6.9 Mb)	7p14.3p15.2 (2.5 Mb)	p.Trp369Ter	p.Asn372His	p.Arg326Gly	p.Ile368Phe
Neurodevelopmental delay	+	Borderline	+	+	+	+	+	+	+	–	–	–	–
Height	Short stature	Normal	Normal at birth	Normal at birth	NR	Low weight at birth, short stature with relative macrocephaly	Low weight, short stature, and low cephalic perimeter	Postnatal short stature	Short stature with relative macrocephaly	NR	NR	Normal	Normal
Frontal bossing	+	NA	NA	NA	NA	+	–	–	+	–	–	–	–
Depressed nasal bridge	+	NA	+	–	+ ^a^	+ ^a^	+	+	+	–	–	–	–
Mild hypertelorism	+	NA	+ ^a^	- ^a^	+ ^a^	NA	+	–	+	–	–	–	–
Long philtrum	+	NA	+ ^a^	+ ^a^	+ ^a^	NA	+	–	+	–	–	–	–
Ear anomalies	Posteriorly rotated and dysplastic	Low-set and malformed	ND	Small and dysplastic	Prominent crus hélix	Posteriorly rotated, low-set, external auditory canal stenosis	Low-set, hypoplastic helix, prominent antihelix and antitragus	Posteriorly rotated, dysplastic, and low-set	Prominent antihelix, upslanted lobule	–	–	–	–
Hand anomalies	Small, bilateral fifth finger clinodactyly	Small, bilateral fifth finger clinodactyly, short distal phalange of second finger	Small, right fourth finger clinodactyly	Small, bilateral fifth finger clinodactyly	Bilateral fifth finger hypoplasia, with single crease on left side	Small, hypoplastic thumb, extension limitation of thumbs, second and third fingers, nail dysplasia, abnormal creases	Small, mesomelic shortening and fifth finger hypoplasia with bilateral clinodactyly, abnormal creases	Small, bilateral fifth finger clinodactyly, small distal phalanges	Small^a^, Thumb and second finger distal phalanges hypoplasia, conic fingers	Small, thenar hypoplasia, abnormal left thumb, right fifth finger clinodactyly, left palmar transverse crease	Small^a^, hypoplastic thumbs, bilateral fifth finger clinodactyly	Hypoplastic thumbs with proximal insertion, fifth finger clinodactyly	Small^a^ hypoplastic thumbs, importantly affected distal phalanges
Feet anomalies	Severe hallux valgus	Hypoplastic thumb, laterally deviated	Small and broad thumb	Small thumb, pes planus	NA	Small feet, thumb hypoplasia, nail dysplasia, abnormal creases	Brachydactyly of toes II–IV with a long and broad hallux, medially deviated	Hallux valgus, short and broad thumb	Small feet and short toes	Small feet, right foot smaller than left foot, bilateral abnormal and small thumbs	Absent thumb	Hallux valgus	Absent thumbs, distal phalanges affected
Genital anomalies	Cryptorchidism, hypospadias	Cryptorchidism, ventral-bowed penis	NA	–	Rectoperineal fistula	Hypospadias	Hypospadias left cryptorchidism and scrotum hypoplasia	–	–	–	Hypospadias	Double uterus and cérvix	Bicornis hypoplastic uterus
Other dismorphias	Facial asymmetry strabism, thin upper lip	Anteverted nares, retrognathia, wide mouth, short uvula, short soft palate	Anteverted nares, broad lips, upslanted palpebral fissures, broad nose	Broad neck, upslanted short palpebral fissures	Depressed supraorbital ridge on the left, maxillary hypoplasia, craniosynostosis, anal atresia	Accessory nipples	Craniosynostosis, short palpebral fissures, epicanthus, ptosis, anteverted nostrils, micrognathia^a^, high palate and soft-palate cleft, short neck, widely spaced nipples	Bifrontal narrowing	Thin lips, micrognathia, short neck	–	–	–	–

Superscript numbers indicate the references

*NR* Not reported

*NA* Not available

^a^ Observed in clinical picture but not referred in the corresponding article; ^b^CGH; ^c^Karyotype

From this analysis, we derived important phenotypic data that might suggest the presence of a microdeletion in the 7p15 region, which include the following: a) Neurodevelopmental delay or intellectual disability present in all patients with the microdeletion. *HOXA1* gene is localized in the minimum shared region and is associated with central nervous system development [22]. Also, in this region is miRNA196b (Fig. 6; green circle) [22], in which the gene target *NRC2* is implicated in neuronal development [23]. b) Short stature is shared by more than half of the patients (5 of 9) with the microdeletion. Both *HOXA5* and *HOXA11* are located within the *HOXA* cluster (Fig. 6; orange circle), dysregulation of which might affect growth and consequently generate short stature [22, 24]. c) Facial dysmorphias that are not present in patients with point mutations are found in 9 out of 9 patients with the microdeletion; the most common finding is dysplastic ears, particularly low-set ears with a prominent antihelix, observed in 8 out of 9 reported patients (in the ninth patient, the ears were not phenotypically described). Other facial features shared by most patients with microdeletion are a depressed nasal bridge and a long philtrum. The *HOXA* cluster is located within the minimum shared region and includes the *HOXA1* and *HOXA2* genes (Fig. 6; blue circle), both of which are related to external ear development. This has been verified in mouse models, where high *Hoxa1* and *Hoxa2* messenger expression levels and low mmu-miR-10a (a modulator of *Hoxa1* expression during ear development) levels have been reported [25].

These genes, as members of the *HOX* gene family, play an important role in embryonic patterning and organogenesis, specifically in the developing region of the rhombencephalus and branchial arch [26–28]. Mouse *Hoxa2* knockout models, as well as pig models with nonsense mutations in *Hoxa1,* have been associated with congenital microtia [29, 30].

Deleted region sizes are variable, so we looked for a minimum shared region in patients with HFGS caused by a 7p15 microdeletion to compare phenotypes and to determine if larger genomic losses cause more dysmorphisms or more severe phenotypes. We found that the patient that had the smallest deletion (2.5 Mb; including the 584,758 bp minimum shared region; Fig. 6) [17], as well as our patient, who has the largest deletion reported so far (12.7 Mb), the classical clinical characteristics of HFGS were sufficient evidence to suggest haploinsufficiency of *HOXA13,* despite the absence of several other genes, responsible of a more severe phenotype, making diagnosis more difficult.

According to the information reviewed in every previously reported patient and the characteristics of our patient, we can suggest that a microdeletion in the 7p15 region must be investigated in every HFGS patient that presents with dysplastic ears, mainly low-set ears with a prominent antihelix, as well as a depressed nasal bridge and a long philtrum. Finally, the patient’s follow-up and management was based on the literature reports [16], and independent of the etiology, the recurrence risk for each pregnancy is 50% for carrier parents and less than 1% for healthy parents.
